# A short history of the toxicology of inhaled particles

**DOI:** 10.1186/1743-8977-9-13

**Published:** 2012-05-06

**Authors:** Ken Donaldson, Anthony Seaton

**Affiliations:** 1 MRC/University of Edinburgh Centre for Inflammation Research, ELEGI Colt Laboratory, Queen’s Medical Research Institute, 47 Little France Crescent, Edinburgh, EH16 4TJ, UK; 2 Environmental and Occupational Medicine, University of Aberdeen, Aberdeen, UK

## Abstract

Particle toxicology arose in order to understand the mechanisms of adverse effects of 3 major particle types that had historically exerted the greatest toll of ill-health—quartz, coal and asbestos. By the middle of the last century rat inhalation studies had been carried out and the pathology documented, but true mechanistic particle toxicology did not really take off until the 1970s when cell culture techniques became available. By the 1980s glass fibres were a major focus of interest and attempts to develop a structure-toxicity paradigm centred on biopersistence. In the 1990s environmental particles dominated the particle toxicology agenda and the cardiovascular system emerged as a target for inhaled particles, raising new challenges for particle toxicologists. We are currently in the era of nanotoxicology where a large and diverse range of new nanoparticles types are under scrutiny.

##  

The aims of particle toxicology are generally those of conventional chemical toxicology, that is to investigate the characteristics of particles that determine their ability to cause harm and the pathobiological mechanism of that harm. Achievement of these aims should enable identification of the best metric to measure particles in order to manage their risk and to intervene in cases where exposure has occurred in order to try to prevent progression. Particle toxicology has problems that other branches of toxicology don’t have, central to which is the variation amongst the particles that are being tested—a problem that continues to confound efforts to make generalisations about their toxicology. This has most recently been found in the sub-specialty of nanotoxicology where the idea that all nanoparticles are equal in toxicity has been slow to counter [1]. Many new recruits to particle toxicology have taken up this area of toxicology because of the flow of funding into nanotoxicology, and we feel it may be helpful to them to know something of the previous history of particle toxicology, of which they may not be aware. While there are other ways in which particles might have adverse effects, our definition of particle toxicology is restricted to inhalation toxicology, meaning the accidental inhalation of particles in workplaces and the environment. A short history of inhalation particle toxicology is therefore our aim, and complements shorter accounts by other authors [2-5]. This is a personal history involving our own perception of the landmarks; a different close observer of the last 40 years of particle toxicology might write a different history. In such a short history it was impossible to cover the work of all the particle toxicologists who have advanced our discipline and we apologise for any such omissions. A lengthier history would be necessary to correct these.

## The first descriptions of dust-related occupational lung disease

**Figure 1:**
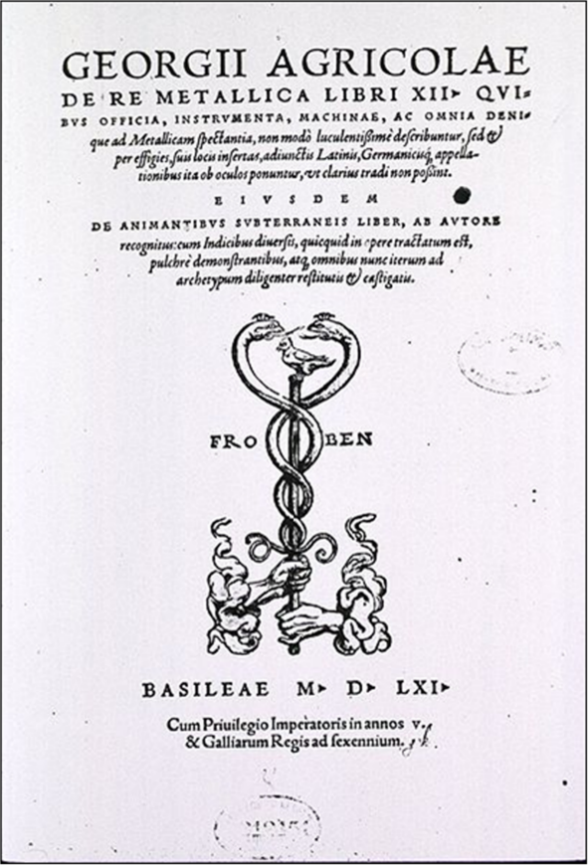
The front page of De re metallica (copyright expired; obtained through (Wikipedia commons).

The first mentions of a link between exposure to dust particles and lung disease are to be found in the 15^th^ and 16^th^ centuries. Within a few years of the invention of the printing press, Georgius Agricola wrote about the theory and practice of metal mining and refining in ‘On the nature of minerals’ (De re metallica) (Figure 1).

Agricola described the theory and practice of mining including finding seams and working them. However he also described how air becomes stagnant in the mines and he described machines to blow fresh air into the mines, early ventilation systems. Along with accidents adversely affecting the miners he also mentioned how ‘*dust which is stirred by digging, penetrates into the windpipes and lungs and produces difficulty in breathing and the disease which the Greeks call asthma*’. It is important to point out that this is not our current use of the term asthma but a generic term for breathlessness. He stated that ‘*In the mines of the Carpathian mountains women are found who have married seven husbands, all of whom this terrible consumption has carried to a premature death* ‘. At that time no distinction was made between different lung diseases but later it was discovered that the miners were at risk from three diseases, silicosis, tuberculosis and lung cancer, all of which are likely to have contributed to their early demise [2]. Agricola did not link the dust exposure directly to the disease, although he recommends a primitive dust mask in the form of ‘veils’ that miners should wear. He is also regarded as the father of occupational hygiene in his description of methods of ventilating mines. Perhaps we should not be too surprised of the lack of simple observational correlation between dust in the air and lung disease since Agricola also wrote an entire book about the demons (De Animantibus Subterraneis) that inhabit mines. These ran about in the darkness chattering and turning out the miners’ lamps but could be vanquished by fasting and prayer!

Paracelsus, best known for his observations on dose in toxicology and for his promotion of the first real cure, mercury, for syphilis, published the first book devoted to occupational disease in 1567. The term contrarian might have been coined for Paracelsus. In an extended ‘gap year’ following graduation in medicine he wandered around Europe meeting all sorts of people from gypsies to midwives and executioners and formed his own brand of folk medicine. On returning in 1526 he became town physician in Basel and lecturer in the University. Paracelsus soon started publicly burning the previous generation’s medical textbooks and giving long, hugely popular lectures recounting his personal experiences; the success of these made him unpopular with his colleagues. Of relevance, he used his experience of smelting plants and mines that he visited during his travels and his work for mines in later years, to develop a theory of pathology in miners’ lungs. He recognised and described the symptoms of miners’ lung disease—dyspnoea, cough and wasting. Like Agricola he failed to make the link to their exposures and instead evoked a theory involving ‘…*the powers of the stars, in that their peculiar characters are boiled out which settle on the lungs in different ways*’ [2]. Concepts of medicine were dominated in those days by belief in disease being inflicted by demons or a consequence of an imbalance of the humours, so these explanations would not have been particularly strange to Paracelsus’ contemporaries.

The person recognised as the father of occupational medicine was however Bernardino Ramazzini, who in 1700 wrote his text ‘Diseases of workers’. His most important insight was to recognise that illness could have an environmental cause. There was a view that disease was sometimes caused by bad air (eg malaria from Italian, *mala aria*) but he extended this to a much wider range of factors. He also was one of the first doctors to concern himself with illness in poor working people. Amongst many insights on occupational diseases, for that time, he discussed ergonomics / bad posture, recommended that there should be good ventilation in dusty trades, spacious rooms with a good draught, and that workers should quit work at the first sign of any lung disease [3].

Despite these early insights, the working conditions of the poor, which included miners and those in dusty trades, were of no interest to legislators until the Industrial Revolution in the early 19^th^ century and little was done to protect workers from danger until the early 20^th^ century. From then on, in the developed world, steps have been taken to reduce exposures in workplaces, by which time exposures to the big three hazards, quartz (crystalline silica), coal and asbestos had exerted a huge toll in terms of particle-related lung disease, and this continues in industrially developing countries to the present day. Sometimes the scientific establishment took the risks rather too lightly. For example J.S Haldane who was honorary Director of the Mining Research laboratory of the University of Birmingham declared in 1923 that ‘*the inhalation of coal dust causes no danger to life but on the contrary gives protection against the development of tuberculosis*’. At that time silica was well known to cause lung disease but the respiratory illness among coal miners was thought to be a consequence of tuberculosis and quartz. Although he had a medical degree, Haldane, a physiologist, was most interested in acute problems such as gassing, explosions and pressure effects, and it was not until pathologists in Cardiff such as Cumming and Gough investigated the lungs of miners in the period 1930–50 that it became apparent that coal dust was toxic in its own right [6,7]. This of course was also partly a consequence of the introduction of medical radiology in that era.

## The ‘big three’ of particle toxicology

Three particle types have been responsible for the greatest amount of lung disease over time and these are dealt with below.

**Figure 2:**
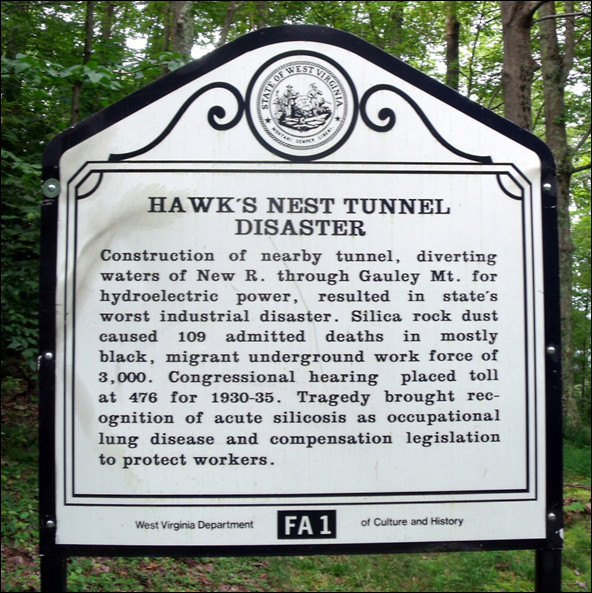
**Hawk’s Nest Tunnel memorial in Ansted, Fayette County, West Virginia, USA.** (From the Historical Markers Database http://www.hmdb.org/marker.asp?marker=34417).

Quartz or crystalline silica is one of the most common minerals in the earth’s crust so that throughout the ages whenever mankind (and they were mostly, but not exclusively men) mined, quarried or worked the surface layers of the earth, they were exposed to quartz dust as the large crystals fractured into respirable particles. This toll of death from silicosis was recognised first in the 18^th^ and early 19^th^ centuries among knife grinders in Sheffield and men who made millstones, but undoubtedly went unnoticed from the time that men shaped arrow heads and axes from flint. Lung cancer in miners, subsequently shown to be due to radon exposure, was first described at the end of the 19^th^ century. In the 1920s the Hawk’s Nest tunnel incident in Gauley Bridge, West Virginia, produced a horrific toll of death and disease (Figure 2). Of the estimated 2,500 workers who worked in the tunnel, 764 died from acute silicosis and an additional 1,500 ultimately developed the disease [8]. Although the employers attempted to cover this up, the details can be read about owing to the fascinating detective work of Martin Cherniack in his book ‘The Hawk’s Nest Incident’ [9]. Unfortunately, silicosis continues to take its toll of human lives, even in developed countries, though to a much greater extent in poorer countries with inadequate regulation of exposed trades.

**Figure 3:**
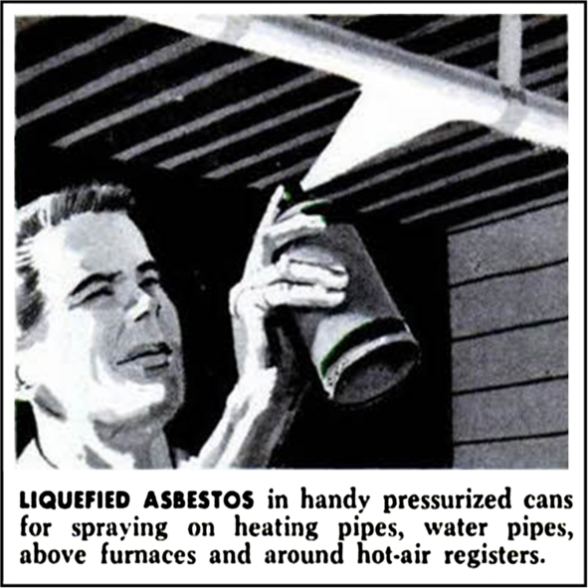
**Advert for ‘liquefied asbestos’ showing ill-advised use of an asbestos spray by a man with no personal protective equipment.** (From Asbestos focus website http://www.asbestosfocus.co.uk/apps/photos/photo?photoid=112479766).

Asbestos was first mined in substantial amounts in the late 1800 s in Canada and was soon being imported in millions of tons per annum from the major producers, Canada, Russia, South Africa and Australia into most developed countries, where it was incorporated into a huge number of products such as cement, tiles, fillers, brake linings, pipes and insulation (Figure 3). Like quartz, asbestos is crystalline and the crystals shatter along fracture planes to release thin fibres that are easily breathed in to the lungs. Asbestos causes a number of lung diseases including asbestosis, a fatal type of interstitial lung fibrosis, and lung cancer but also affects the pleura for reasons that are becoming better understood [10]. In the pleura it gives rise to pleural fibrosis, plaques and mesothelioma, the most feared of the asbestos diseases. Pleural effects in humans, especially mesothelioma, have only been seen with asbestos and one other long fibre-shaped particle, erionite, that is not part of the asbestos family; pleural effects are not seen with other particles except in very advanced silicosis and mesothelioma is only seen with fibres. Mesothelioma is a slow-growing but highly malignant tumour that causes death usually within a year or two of diagnosis, with no known cure. Although asbestos is no longer used in most developed countries, in many countries it still is, and exposure still occurs from its removal or disturbance. In Western countries thousands of mesothelioma deaths occur every year owing to the use of asbestos until the 1980s and this is likely to continue for decades in most countries, particularly where amphibole asbestos has been used [11].

**Figure 4:**
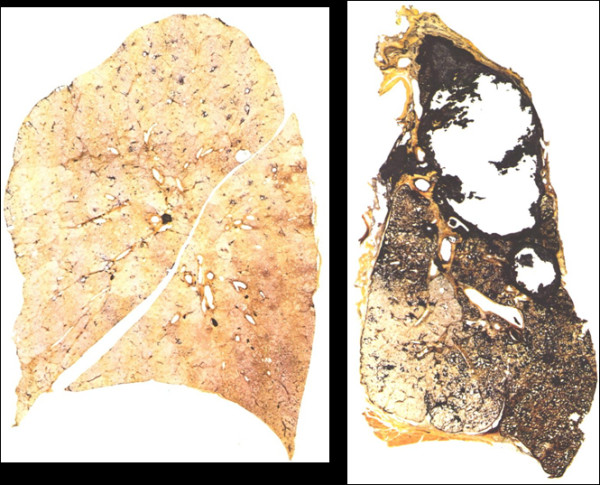
**Gough Wentworth paper- mounted thick sections of the entire lung of a non-smoking non-miner (left) and a coal miner with cavitating progressive massive fibrosis (right).** The left hand lung shows some centri-acinar dust accumulations in the upper parts related to air pollution but is otherwise normal. The miner’s lung shows the dark pigmentation of coalmine dust with emphysema and almost total destruction of the upper lobe with apical pleural thickening. (with permission from Ingelheim portfolio 11 Lung sections).

Coal causes a form of pneumoconiosis (coal-worker’s pneumoconiosis) that is usually less severe than silicosis. Coalmine dust comprises mostly low toxicity particles containing a variable mixture of carbon, quartz and silicates, and coalworker’s pneumoconiosis is a lung disease caused by large accumulations of coal dust-laden macrophages with a modest local tissue reaction of mild fibrosis usually accompanied by emphysema (Figure 4). In a significant proportion of individuals it develops into progressive massive fibrosis, probably related to accumulation of very high lung burdens caused by the dust-induced inflammatory reaction in draining lymph nodes blocking clearance [12].

In the middle of the 20^th^ century the UK National Coal Board’s ‘Pneumoconiosis Field Research’ developed an integrated approach to understanding the link between coalmine dust exposures and coal worker’s pneumoconiosis, using epidemiologists, hygienists, pathologists and radiologists. Radiographs of coal workers were used to monitor prevalence of the disease and the risk of this was related to measured dust exposures of the miners. In addition, the extent of the disease in many of the miners’ lungs was assessed at autopsy. The ambitious aim was to determine the elements of the coalmine dust (quartz, clays, coal itself) that were responsible for disease. This analysis was carried out in the Institute of Occupational Medicine in Edinburgh. This huge undertaking initially involved the development of a sampler (MRE114A) that was critical for determining exposures to respirable dust. In fact when the huge amounts of data were brought together, including more than 30,000 chest radiographs alone, the conclusion was clear—no single component of the coalmine dust mix was responsible and the total respirable dust was the metric that was best related to disease development. The exposure-response relationship calculated by Michael Jacobsen and colleagues led to agreement between trades unions, employers and government on a coalmine dust standard of 7 mg/m^3^ of respirable dust [13]. This standard was successfully introduced to the UK coalfields and its application was associated with a much reduced prevalence of CWP. The USA took the same data and developed their own standard of 2 mg/m^3^ respirable dust (measured differently from the UK) which also produced enormous advantages in worker protection [14].

Throughout the middle and later twentieth century there were similar attempts to determine safe levels of asbestos and quartz, with varying success in terms of getting the airborne levels lowered by adoption of tighter standards over time This was often exacerbated by resistance from industries to admitting the harmfulness of the dusts and their reluctance to invest in costly dust suppression equipment.

## The rise of particle toxicology

**Figure 5:**
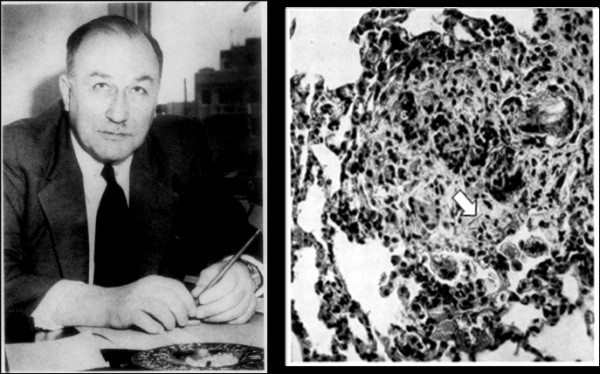
**(Left) Earl Judson King (1901–1963) and (right) a figure from his 1946 paper**[20]**of a fibrotic lung lesion in a rat exposed to asbestos fibre clearly showing a ferruginous body (a coated asbestos fibre; arrow) in the lesion and multinucleate giant cells.** (Used with permission J. Clin. Pathol).

Case reports and epidemiology usually have made the first association between exposure to a dust and development of disease, and epidemiology can explore the exposure-response relationship that is so necessary for standard setting. However toxicology can also make a valuable contribution to prove that an exposure is indeed the cause of a disease using Koch’s postulates type of arguments. Additionally, as discussed later it can be used to analyse the properties of particles that make them harmful, something that is difficult to do in the mixed exposures in human populations, and can thus provide information on mechanisms. E.J. King can justifiably be seen as the first major UK particle toxicologist, although he was a Canadian by birth [15]. Between the middle 1940s and the early 1960s he systematically demonstrated silicosis in rats exposed to quartz and mixed dusts [16-18], its modification with aluminium compounds [19], the effects of asbestos on rat lungs [20] and the effects of particles on cells in culture [21]. Early theories of the mechanisms of quartz toxicity divided into 2 camps—the solubility theory and the mechanical theory. It is interesting to note that the Canadian group of Denny and co-workers, in pursuit of the solubility theory, had demonstrated a protective effect of aluminium on silicosis in rabbits in the late nineteen thirties [22] (Figure 5).

The solubility theory was based on the idea that soluble silicic acid released from the quartz surface was the harmful component, whilst the mechanical theory implicated the sharp and angular quartz particles as mechanically irritating the lungs [23]; neither of these paradigms has survived to present day particle toxicology.

The first real mechanistic particle toxicology work on asbestos had an emphasis on the quantification of dose, a vital step, and was carried out by Dr Chris Wagner. Whilst working as a young pathologist in Johannesburg, Wagner had noted pleural mesothelioma, a very unusual tumour, in workers from the crocidolite mining area in the Cape Province of SA and reported this in 1960 [24]; this established the first clear link between asbestos exposure and its hallmark tumour, though earlier reports had noted a connection. He then came to the UK to work in the MRC pneumoconiosis unit in Llandough, Wales. By 1965 Wagner began publishing an influential series of papers beginning with asbestos deposition and retention, the first emphasis on the modern concept of particle dosimetry [25]. He went on to demonstrate mesothelioma production in rats after exposure to crocidolite [26] and then carry out a number of long term rat inhalation studies that set a new standard for this type of study [27-30] exploring the role of asbestos types and their mechanisms. Similar work was carried out in the Institute of Occupational Medicine in Edinburgh led by Dr John M.G. Davis from the mid-seventies onwards. The Davis group went on to produce a sequence of important papers on the relationship between asbestos characteristics and pathogenicity [31-34], many of which were inspired and executed by Rob Bolton, whose PhD had been supervised by John Davis in the early seventies. In the USA at the same time, Merle Stanton at the National Cancer Institutes was studying mesothelioma induction by asbestos in rats using an implantation technique onto the visceral pleura. The Wagner, Davis and Stanton groups all identified critical roles for fibre type and length, igniting a substantial amount of research on the role played by length, fibre type and biopersistence in toxic potency that continues to the present day and which has defined the first real structure:toxicity model for a pathogenic dust [35-37]. In addition this work was important from a practical point of view in terms of defining exposure, since it allowed formulation of the fibre-counting rules and led to the Walton Beckett graticule [38] which assists in the identification of respirable fibres. This enabled a scientific method of fibre counting and thus regulation of the exposure to fibres in workplace air.

**Figure 6:**
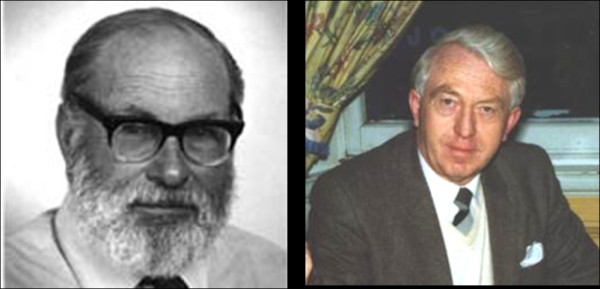
**Left Dr Chris Wagner (1923–2000) MRC Pneumoconiosis Research Unit, Llandough; (From the website of the Mesothelioma Interest Group **http://imig.org/about/wagner-award-recipients-2**). Right Dr John M.G. Davis (1935–2011) Institute of Occupational Medicine, Edinburgh. Property of KD)**

Following on from Stanton’s work, in Germany Friedrich Pott and co-workers championed the use of the intraperitoneal test as a sensitive assay for the potential of fibres to cause mesothelioma. The use of the intraperitoneal test has, however, proved a controversial issue, although it has become established in European regulation (Figure 6).

### Particle toxicology comes of age

By the late seventies the pace in particle toxicology was quickening and there was a burgeoning community of particle toxicologists, heralding the dawn of cellular toxicology and applying its techniques to cells in culture as the new methods became available. The first in the series of meetings, still ongoing, then named ‘In vitro effects of mineral dusts’ was held in 1979 (see [5] for the history of this meeting). This era witnessed the first attempts to find predictive tests for toxic particles, although the understanding of the key pathological processes such as fibrosis and cancer was not sufficiently advanced for assays to be soundly based on an understanding of the molecular pathology. In fact the Proceedings of the first meeting, held in Cardiff in 1979 [39], were dominated by papers measuring cell death, since that was about all that could be quantified! A few standard methods came into use that still operate now—e.g. the use of a control, harmless particle like TiO2 and a positive particle like quartz to allow benchmarking of any unknown particles under test. Organ culture was also used and [Brooke T. Mossman 40] in the University of Vermont used hamster trachea to demonstrate the effects of asbestos and went on to investigate the molecular mechanism involved in asbestos effects over the next 30 years, pioneering the oxidative stress theory and demonstrating the molecular pathways by which particles influence cells. By the early eighties Arnold Brody in the National Institute of Environmental Health Sciences in North Carolina was using novel and creative electron microscopy approaches to open up the ‘black box’ of the lung with regard to the early responses to pathogenic dust inhalation. Brody demonstrated that there was high efficiency deposition of inhaled respirable fibres in the centri-acinar and alveolar duct regions of rats [41,42] leading to high localised dose and a rapid pathobiological response [43,44]. David Warheit, later of DuPont, worked in NIEHS at this time and was involved in these ground-breaking studies [45], which were the first to demonstrate the rapidly evolving response to a pathogenic dust in this sensitive area, identifying the mechanism of macrophage recruitment to sites of fibre deposition, followed by proliferation of epithelial and other cells and prototypic fibrotic lesion development at the first alveolar duct bifurcation.

In the meantime, in the eighties and nineties new particles came along to occupy particle toxicologists as the ‘big three’ became better understood. These were the man-made mineral fibres (MMMF), later renamed man-made vitreous fibres (MMVF) and even later renamed synthetic vitreous fibres (SVF). Some of the SVF were seen as direct replacements for banned asbestos, to fill the gap in the industrial need for fibres, but were still fibres and so suspicion arose that asbestos might be being replaced by a similar hazard. These SVF, glasses and ceramics of various sorts that are spun or blown out into fibres, are used for insulation and numerous other purposes. Many studies carried out comparisons between asbestos and the vitreous fibres, culminating in the emergence of ‘biopersistence’ as a key explanatory attribute for discriminating between carcinogenic and non-carcinogenic long fibres [46,47]. This era culminated in the IARC Working party on the carcinogenesis of these materials in 2001 [48]. The outcome of these deliberations was that biopersistence dominated; the non-biopersistent SVF were exonerated as carcinogens and the more biopersistent ones were classified as carcinogens. A side-effect of the discovery of biopersistence as a key factor in SVF pathogenicity was the uncovering of biopersistence as the explanation for differences between the more harmful amphibole asbestos and the less harmful chrysotile asbestos, the latter having been shown to be less persistent both *in vitro* and *in vivo*. This period showed the first full application of a number of tests in particle toxicology that are now standard, including genotoxicity tests such as the micronucleus assay and the rise of the measurement of cytokines and use of bronchoalveolar lavage to study lung inflammation and understand mechanisms of inflammation development. It also witnessed the development of a benchmark standard that still pertains today for particle/fibre inhalation studies in the form of the studies by the Research and Consulting Company (RCC) on SVF [49,50]. These emphasised exposure to respirable diameter fibres of the length found in workplaces, lung burden studies, non-destructive aerosolisation, accurate bimodal fibre counting, bronchoalveolar lavage (BAL) etc. Similar but smaller—scale inhalation studies performed in Edinburgh under the auspices of the Colt Fibre Research Programme in the Institute of Occupational Medicine in the early nineties came to the same conclusion regarding the central role of biopersistence in fibre toxicity [51-53].

## Ambient particles

By the middle nineties, with SVF receiving less research attention in the wake of the IARC rulings, environmental particles (PM_10_) became of increasing interest for particle toxicologists. The prevention of coal burning in urban areas by the enactment of the Clean Air Acts in the UK and elsewhere, had largely eliminated the dense winter smoke fogs that saw epidemics of thousands of deaths, as in the ‘great smog’ of 1952 in London, and there was a feeling that the problem of pollution in cities had been largely solved. However, epidemiological studies of huge populations, entire cities, began to show correlations between death rates and concentrations of particles and sulphur dioxide in the air. The studies revealed a short-term relationship between these two variables in ‘time-series’ studies and more long-term effects of living in polluted areas in longitudinal studies. The most famous of the latter type was the study which examined six US cities that had differ*e*nt average levels of particulate air pollution and showed that death rates in each city were related to the concentrations of the particulate air pollution [54]. It seemed that, although the visible soot-laden air that had characterised cities since earliest times was no longer evident, the increase in traffic had brought a new kind of air pollution derived largely from combustion in vehicle engines.

By the early 1990s it was apparent that although the mass of particles in air pollution had fallen dramatically in industrial societies in the West, effects on health in terms of heart and lung disease were still apparent, though by now the predominant source of the pollution had changed from coal burning to vehicles. The introduction of new statistical techniques made it possible to demonstrate that relatively small fluctuations in pollution concentrations were associated with similarly small fluctuations in health effects, from heart attack deaths to consultations with doctors for exacerbations of asthma. Whether these effects derived from particles or toxic gases was a major issue, particularly in terms of what to regulate, but the most remarkable fact was the low concentrations at which the associations were observable. It was notable that although as one might expect respiratory illness occurred, the greater number of deaths and hospitalisations occurred from heart disease, usually heart attack [55]. This fact, that an organ distant from that in which the toxic agent was deposited was the one most affected seemed paradoxical and was an obvious challenge to toxicology. As research advanced, particles were seen to act by a variety of pathways to induce inflammation and oxidative stress in the lungs [56-58], making the link to exacerbations of airways disease and even possibly lung cancer, though the epidemiology on this was, at the time, weak. The question as to how pulmonary deposition of extremely low concentrations of mainly carbon particles might cause heart attack was first addressed in a hypothesis paper that emanated from a discussion in 1994 between the authors, David Godden and Bill MacNee. Seaton, following a conversation with Robert Waller in 1993, chanced upon a paper on seasonal plasma fibrinogen fluctuations and it immediately occurred to him that pollution exposure might act through such an amplifying mechanism that might lead to cardiovascular deaths by promoting clotting in the coronary arteries. The question of low dose was addressed by thinking in terms of numbers rather than mass. It had been known since the 19^th^ century that air pollution contained many very small particles, and this was demonstrated by the Lawther and Waller group in the 1960s. The highly original work of Ferin and Oberdorster and colleagues had shown unexpected increases in toxicity of ultrafine particles, or what are now generally characterised as nanoparticles [59]. Thus we were able to propose that ‘…*inflammation provoked by ultra-fine particles, in addition to promoting exacerbations of lung disease, has an additional effect on the coagulability of blood, increasing the susceptibility of individuals to acute episodes of cardiovascular disease…*’ [55]. This brief but highly–cited paper preceded a huge research effort and clear evidence of a link from the lungs to the blood vessel wall and the atherosclerotic plaque subsequently emerged in many studies, for example using ApoE mice [60] and human chamber studies [61]. In fact a persuasive mechanism has emerged based on relatively low exposures of human subjects to diesel exhaust particles (DEP) inducing lung inflammation and oxidative stress. These indirectly affect cardiac blood flow, [62] the associated endothelium [63,64] and the clotting system [65] in ways that favour atherothrombosis. These data have emerged from a fruitful collaboration between Edinburgh University (Professor David Newby, Dr Nick Mills), University of Umea in Sweden (Professor Thomas Sandström, Professor Anders Blomberg) and the RIVM in the Netherlands (Professor Flemming Cassee). The overall approach utilises cells, rodent and human studies in a highly innovative model program aimed at determining the link between pulmonary deposition of diesel particles and atherothrombosis. Whilst indirect effects of inflammatory mediators and oxidants from the lung means that a role for translocation of the combustion-derived nanoparticles to the blood in driving these events seems to be receding, translocation still remains an open question.

This emphasis on diesel exhaust nanoparticles is not meant to detract from the likelihood that the fine and coarse fractions may have some role in the adverse effects of PM depending on the origin of the particles and their chemical composition e.g. [66].

## Nanoparticle toxicology /nanotoxicology

**Figure 7:**
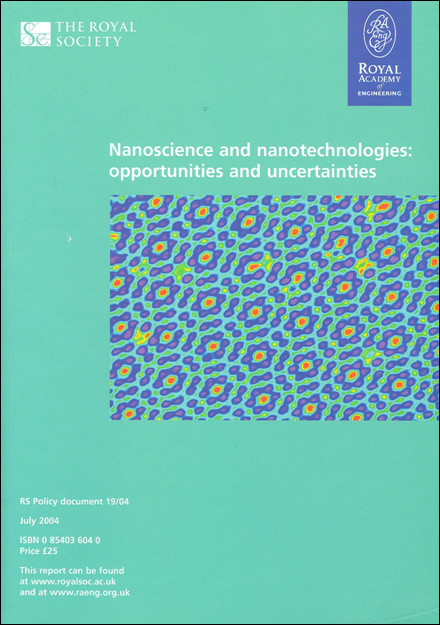
Royal Society and Royal Academy of Engineering report ‘Nanoscience and nanotechnologies: opportunities and uncertainties’ published in 2004.

The 1990s saw extraordinary advances in technology which gave rise to the possibilities of engineering materials at sub-micron scale and thereby producing new materials with specifically enhanced or altered properties, nanotechnology. It was foreseen that this would open up a whole new world of industrial and commercial applications, but at the same time concerns began to be expressed that some of these altered properties might imply altered or enhanced hazard to humans or the natural environment. The UK government asked the Royal Academy of Engineering and the Royal Society to consider these opportunities and uncertainties. At the same time the term nanotoxicology was coined in a commentary by Robert Service in the journal Science in 2004 [67] and a number of European particle toxicologists, led by one of us (KD), laid out what amounted to a manifesto on this new area of particle toxicology in an Editorial in the UK journal Occupational and Environmental Medicine entitled ‘Nanotoxicology: A new frontier in particle toxicology relevant to both the workplace and general environment and to consumer safety’ [68]. This concern about new manufactured nanoparticles (MNP) being developed in industry was predicated on the experience of particle toxicology which had shown that surface area and reactivity were important determinants of toxicity and which had suggested that combustion-derived nanoparticles were a main factor in ambient particle toxicity (see [4]for a fuller history and the origins of nanotoxicology). The possibility that new nanotechnologies might produce other materials posing similar hazards to asbestos or air pollution particles was a powerful driver in raising the concerns that were crystallised in the influential report of the UK Royal Academy of Engineering and Royal Society [69] (Figure 7).

There has been an enormous amount written on the issue of nanotoxicology, much of it generating more heat than light, but it remains at the time of writing the primary concern for particle toxicologists, and the primary source of funding. Carbon nanotubes for example have been found to be highly fibrogenic in the lungs [70] and if they are in a fibrous morphology, to have the potential to produce asbestos-like effects in the peritoneal and pleural spaces [71,72]. These toxicological findings have already influenced risk assessment and standard setting in relevant workplaces.

It is interesting to observe that prior to the development of manufactured nanoparticles (MNP), concern that a particle could have an adverse effect tended to develop in the following way:

1. a single or a few cases were reported in a workforce who handled and were exposed to a specific particle.

2. toxico-pathological studies suggested a causative relationship between the material inhaled and disease.

3. epidemiological investigations demonstrated the nature of the relationship and possibly estimated an exposure-response relationship.

4. toxicologists confirmed and further enhanced understanding by controlled animal experimentation and explored the molecular mechanism using cell models.

5. particle toxicologists examined the role of the particle structure in the toxicity.

It is striking however, that for MNP this process is working the other way round. Many MNP under study are in general not yet being handled in large enough quantities or for long enough for disease to emerge. Therefore toxicologists are trying to predict the diseases or pathological reactions that might arise from exposure to MNP before there is any evidence that they are occurring or will occur. This would seem to be an improvement on waiting to ‘count bodies’ on the introduction of a new hazard. However, it does raise the possibility of false positives especially if significant exposures do not occur. This is new territory and the predictive power of the assays we use are being themselves tested to the breaking point while exposure data need to be integrated with the toxicological data to decide risk—not an easy process. It remains to be seen how this will all resolve and which if any nanomaterials represent significant risk.

### Cigarette smoke

Cigarette smoke certainly exceeds ‘the big three’ in terms of the ill health that it has caused but, apart from a smattering of papers in the early years, particle toxicology has not embraced cigarette smoke. This has become even more marked in the last 20 years, when the ability to publish toxicological (as opposed to epidemiological) research on cigarette smoke effects has been greatly impaired and those studying cigarette smoke, or taking funds for that purpose, have become to an extent marginalised by the scientific community. To our knowledge no papers on cigarette smoke have ever appeared in the journals Particle and Fibre Toxicology nor in Nanotoxicology and very few have ever appeared in the Inhaled Particles series or the Particle Toxicology series. From the point of view of toxicology the relationship between the main pathological consequences in the lungs—cancer and COPD—and particulate versus the gaseous and organic phases is not resolved and particles themselves may not play the primary role. Cigarette smoke remains a marginal issue for particle toxicologists and one that has complicated political and social issues related to it.

#### The dose in particle toxicology

It seems important to finish on ‘dose’, the most pertinent entity in toxicology. In particle toxicology, good dosimetry and the ‘biologically effective dose’ have proved elusive. Tissue burdens, including mass balance toxicokinetics, are necessary in order to understand the complete picture of any toxin, especially the role of the translocated dose that reaches beyond the portal of entry and its mechanism and consequences. This was seldom done in an integrated way in particle toxicology until the emergence of a key German/USA collaboration. This collaboration, between Gunter Oberdörster and Wolfgang Kreyling has been central to the current view of particle dosimetry that dominates the discipline. Oberdörster worked in the Fraunhöfer in Hanover but relocated to the University of Rochester in the 1980s where there was already strength in depth in particle toxicology through the work of Juraj Ferin and Paul Morrow. Kreyling was, and still is, employed at the Helmholtz near Munich with a large lung dosimetry group which originally included particle toxicologists such as Helmut Greim and Joachim Heyder. Kreyling’s strength in mass balance particle toxicokinetics was matched by Oberdörster’s insightful and skilful command of toxicology and between the two of them they virtually invented modern particle dosimetry.

This has an emphasis on quantitative aspects of particle dose including the dose rate, the need to understand deposition, clearance and translocation and their relation to pathobiological mechanisms and the response seen at the tissue and cell level, in the lungs and beyond.

#### Postscript

The era of particle toxicologists who originally saw coalmine dust, quartz and asbestos as the big problem and were involved in the invention of particle toxicology as a discipline is almost over. Nanotoxicology now dominates particle toxicology and for many people nanoparticle toxicology is particle toxicology. In fact the issues surrounding the big three have never been fully resolved and there remain many unanswered questions in conventional particle toxicology. For example, why is erionite so potent a cause of mesothelioma? What is the mechanism of quartz toxicity? Why do only rats get overload? The avalanche of funding that flowed into nanotoxicology will end soon, new particles and new issues will arise, and many questions remain in conventional particle toxicology, so there will always be a place for particle toxicologists. Multidisciplinarity has always been a feature of particle toxicology and this has been enhanced with the rise of nanotoxicology. The emphasis placed on particle characterisation in nanotoxicology has increased the need for particle toxicologists to work with chemists, physicists and material scientists. It is hoped that the outcome of this is that we edge nearer to a structure-toxicity relationship, the “philosopher’s stone” of particle toxicology. The realisation of a full structure-toxicity model would mean that determination of the physicochemical properties of a new particle would enable its toxicity to be predicted without recourse to animal or cell experiments.

While particle toxicology remains a niche area it now has its own specific journals—Particle and Fibre Toxicology and Nanotoxicology; other nanomaterial journals also accept papers on nanotoxicology whilst particle toxicology papers occasionally make it into the general molecular medicine literature. It is to be hoped that having our own journals helps our general profile and that the Impact Factors for these journals continues to increase.

Those new to particle toxicology are encouraged to consult the series of Proceedings of the rolling meetings on particle toxicology that is mentioned earlier in this review (see review [5]) to gain more detailed insight into how particle toxicology has developed, if they are interested. If you are reading this then you are probably contributing to what will become the history of particle toxicology in the future and we hope that this short history has helped to place the subject in context.

## Competing interests

Both authors have no competing interests to declare except to have been involved in some of the most recent history described.

## Authors’ contributions

KD and AS contributed equally to the manuscript, drawing on their experience of the history of particle toxicology. Both authors read and approved the final manuscript.
